# Photocatalytic Nanofiber Membranes for the Degradation of Micropollutants and Their Antimicrobial Activity: Recent Advances and Future Prospects

**DOI:** 10.3390/membranes11090678

**Published:** 2021-08-31

**Authors:** Mandla B. Chabalala, Nozipho N. Gumbi, Bhekie B. Mamba, Mohammed Z. Al-Abri, Edward N. Nxumalo

**Affiliations:** 1 Institute for Nanotechnology and Water Sustainability, College of Science, Engineering and Technology, University of South Africa, Roodepoort 1709, South Africa; mandla.chabalala@gmail.com (M.B.C.); gumbinn@unisa.ac.za (N.N.G.); mambabb@unisa.ac.za (B.B.M.); 2 State Key Laboratory of Separation Membranes and Membrane Processes, National Centre for International Joint Research on Membrane Science and Technology, Tianjin 300387, China; 3 School of Materials Science and Engineering, Tianjin Polytechnic University, Tianjin 300387, China; 4 Nanotechnology Research Centre, Sultan Qaboos University, P.O. Box 17, Al-Khoudh 123, Oman; m.alabri@gmail.com; 5 Department of Petroleum and Chemical Engineering, College of Engineering, Sultan Qaboos University, P.O. Box 33, Al-Khoudh 123, Oman

**Keywords:** nanofiber membranes, photocatalysis, antimicrobial properties, micropollutants, wastewater treatment

## Abstract

This review paper systematically evaluates current progress on the development and performance of photocatalytic nanofiber membranes often used in the removal of micropollutants from water systems. It is demonstrated that nanofiber membranes serve as excellent support materials for photocatalytic nanoparticles, leading to nanofiber membranes with enhanced optical properties, as well as improved recovery, recyclability, and reusability. The tremendous performance of photocatalytic membranes is attributed to the photogenerated reactive oxygen species such as hydroxyl radicals, singlet oxygen, and superoxide anion radicals introduced by catalytic nanoparticles such as TiO_2_ and ZnO upon light irradiation. Hydroxyl radicals are the most reactive species responsible for most of the photodegradation processes of these unwanted pollutants. The review also demonstrates that self-cleaning and antimicrobial nanofiber membranes are useful in the removal of microbial species in water. These unique materials are also applicable in other fields such as wound dressing since the membrane allows for oxygen flow in wounds to heal while antimicrobial agents protect wounds against infections. It is demonstrated that antimicrobial activities against bacteria and photocatalytic degradation of micropollutants significantly reduce membrane fouling. Therefore, the review demonstrates that electrospun photocatalytic nanofiber membranes with antimicrobial activity form efficient cost-effective multifunctional composite materials for the removal of unwanted species in water and for use in various other applications such as filtration, adsorption and electrocatalysis.

## 1. Introduction

Micropollutants found in water systems continue to pose a threat to living organisms. These micropollutants are classified as organic (e.g., herbicides, pesticides, dyes, pharmaceuticals, phenols, polyaromatic hydrocarbons, endocrine-disrupting chemicals and natural organic matter) [1,2,3], inorganic (such as heavy metals, mineral acids, metal compounds, and cyanides) [4,5,6], and biological pollutants (such as parasites, bacteria, pathogens, and viruses) [7,8]. Pollution of water by micropollutants can occur naturally and/or through the release of contaminants either intentionally or accidentally due to human activities such as mining, manufacturing, and agriculture. Recently, research has given much attention to the treatment and removal of strong recalcitrant pollutants such as phenols, alcohols, nitrogenous compounds, sulphur compounds and dyes that are mostly hydrophobic and resistant to biodegradation [9,10].

**Figure 1:**
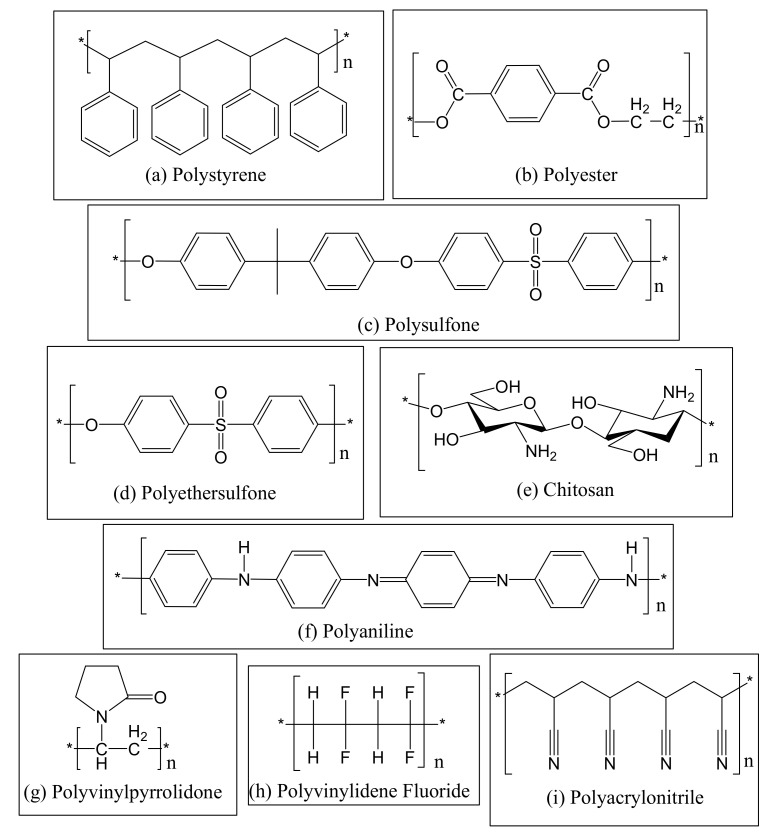
Chemical structures of different types of polymers that are often used in the fabrication of nanofiber membranes. The asterisk (*) indicates that the structure is continuous.

The fabrication and application of electrospun nanofiber membranes embedded with photocatalytic and antimicrobial nanomaterials have been at the forefront of the research in recent times [11]. Indeed, polymers such as polystyrene, polysulfone, polyethersulfone, polyester and polyacrylonitrile (Figure 1) have often been used in the production of nanofiber membranes with desired properties for various applications via an electrospinning process or other desired methods such as polymer blending and sea/island cross-section conjugation [12,13,14]. These polymers can be electrospun on their own or co-polymerised with other polymers depending on the required application. Polymers are often coupled with others to produce polymer products with superior properties compared to mono-polymer counterparts [14].

Polystyrene (PS) is one of the widely used polymers in nanofiber production. It exudes high electrical resistance and low dielectric loss. It is stiff and brittle [12], cheap, easy to handle, and displays a good balance of electrical, mechanical and chemical properties [15]. Polystyrene also finds application in heavy duty polymer materials such as in containers and packaging of electronic goods, ion-exchange materials, membranes, sensors and filtration due to its ease of fabrication, dimensional stability and contact efficiency [16]. However, it is hydrophobic and this limits its full use in water treatment applications [17]. On the other hand, polyester (PET) is used to synthesise nanofibers, membranes and nanotubes for various applications [13]. Natural PETs have advantages such as low cost, ease of separation, low density, CO_2_ sequestration, biodegradability and enhanced energy recovery compared to synthetic PET [18,19,20]. Beside nanofibers for water treatment, PET resins have been reinforced with natural fiber to make materials such as engine covers [20]. Commercially available bio-PET include poly(lactic acid) (PLA), polycaprolactone (PCL) and poly(ester amide) (PEA), among others [18].

In the class of thermoplastics, polysulfone (PSf) has been extensively studied in membrane technology and nanofiber fabrication. PSf materials have good heat-ageing resistance, high mechanical property, thermal and chemical stability [14,21]. PSf based materials have been widely applied in food processing, biotechnology, and water treatment [14]. Polyethersulfone (PES) is another thermoplastic used in various material preparation processes as a modifier or as the main polymer. PES is a synthetic polymer that is non-degradable and biocompatible, oxidative, thermally stable, and exhibits hydrolytic stability, good film-forming and excellent mechanical properties [22]. It has found tremendous application in the fields of filtration, tissue engineering, bioreactors and haemodialysis [22,23,24]. 

Polyacrylonitrile (PAN) is one of the most widely used polymers for fabricating different types of membranes due to its excellent properties, which include ease of electrospinning, high solvent resistance, high mechanical strength, enhanced thermal and chemical stability, good membrane forming ability, biocompatibility and ease of modification [25,26,27,28,29]. PAN is also the predominant precursor to produce nano- to microscale carbon fibers due to its high fiber yield, high mechanical strength and elastic modulus tailoring [30,31]. The PAN polymer fibers are subjected to thermal treatment where they undergo carbonisation and graphitisation at the desired temperature, and are subsequently transformed into carbon fibers [31,32]. Other polymeric materials that are used for the fabrication of different types of membranes, including nanofiber membranes include chitosan, polyaniline, polyvinylpyrrolidone, and polyvinylidene fluoride [33,34,35,36] as shown in Figure 1.

Polymeric membranes are however susceptible to drawbacks such as fouling, poor flux, poor rejection, and short lifespan. As a result, efforts have been made to eliminate or reduce the occurrence of these setbacks and produce composites with superior properties. Methods of modification include additive blending, chemical treatment and surface grafting [37,38]. The commonly practiced methods include the blending of two or more polymers, incorporation of nanoparticles or both, blending with photocatalysts, depending on the desired application and properties. Blending polymers and/or incorporation of nanoparticles may enhance or suppress the intrinsic properties or even add new or novel properties to the bare polymer material [39,40,41]. 

**Figure 2:**
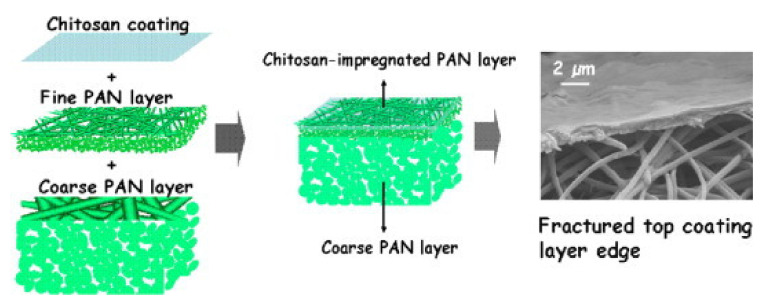
An example of a composite nanofiber membrane consisting of an electrospun polyacrylonitrile (PAN) layer coated with a chitosan layer. Reprinted from [42] with permission from Elsevier.

Figure 2 shows an example of nanofiber membranes produced via electrospinning using fine and coarse polyacrylonitrile polymer coated with chitosan [42,43]. The nanocomposite membranes were fabricated with three layers: (I) nanofiber polyacrylonitrile coarse layer which was coated with (II) fine nanofiber polyacrylonitrile and finally with (III) chitosan [42,43]. It is demonstrated that traditional flat-sheet membranes can be coupled with nanofiber membranes to produce composite membranes with enhanced adsorption capacity, increased surface area to volume ration, and ease of modification properties. 

**Figure 3:**
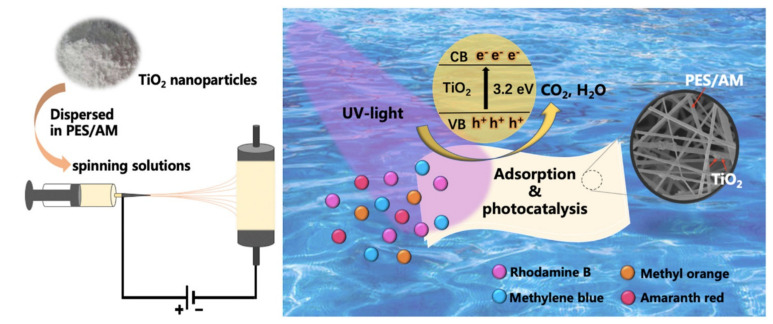
Fabrication of Polyethersulfone (PES)-TiO_2_ nanofiber composite membrane via electrospinning as well as simultaneous adsorption and photodegradation of micropollutants. Reprinted from [44] with permission from Elsevier.

On the other hand, Figure 3 demonstrates the electrospinning of PES nanofiber membranes infused with TiO_2_ nanoparticles for simultaneous adsorption and photodegradation of water pollutants (organic dyes) as reported by Xu et al. [44]. The TiO_2_-PES nanofiber composite membrane was prepared via a combination of blending modification and electrospinning technology. Adsorption activity was reported to be via electrostatic attraction. Photodegradation studies resulted in the elimination of residual toxins completely and adsorption active sites were regenerated by continuous UV irradiation without any other treatments. Recyclability enhancement of over 95% even after 5 cycles was obtained [44]. The incorporation of TiO_2_ nanoparticles to the adsorption membrane introduced photocatalytic and self-cleaning properties, rendering the membrane more efficient and highly recyclable. In latter sections of this review, various other types of polymer-photocatalyst nanofiber membranes with specific examples are discussed in comprehensive detail.

While this review is focused on nanofiber membranes infused with photocatalytic and antimicrobial nanoparticles, Nasreen et al. previously reviewed the general advancement of modification and application of electrospun nanofiber membranes in water treatment. The review emphasizes the importance of nanofiber membrane modification for enhanced efficiencies. Modifications discussed include surface modification (improved selectivity and hydrophilicity) and interfacial polymerization (improved strength, chemical/thermal stability and introduction of selective barrier layer, porous support and/or maintaining strength and configuration). The specific application of these nanofiber membranes covers removal of heavy metals, microbial removal, and desalination [45]. The review paper at hand therefore reviews the current progress made on the development and application of electrospun polymer nanofiber membranes embedded with photocatalytic and antimicrobial nanoparticles for various types of applications with a special focus on the removal of micropollutants from wastewater. In subsequent sections, we therefore discuss (i) these photocatalytic nanofiber membranes with respect to their preparation methods and performance properties, (ii) antimicrobial activities of nanoparticle-infused nanofiber membranes towards microorganisms found in water systems and (iii) various other applications of hybrid photocatalytic membrane processes. 

## 2. Photocatalytic Electrospun Nanofiber Membranes

**Figure 4:**
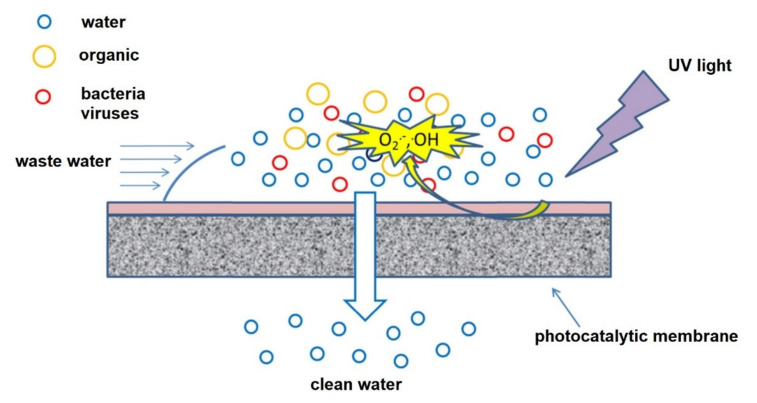
Schematic illustration of how a photocatalytic membrane operates with the photocatalytic layer on top degrading pollutants and membrane filtering the remaining pollutants. Reprinted from [50] with permission from Elsevier.

Photocatalysis is one of the most efficient treatment methods for wastewater containing different types of pollutants. Photocatalysts in their powder form produce tremendous results, often up to complete mineralisation; however, they have drawbacks such as poor recovery and secondary contamination due to leaching [46]. As a result, blending photocatalysts with support materials such as polymers and electrodes has been implemented and well-investigated [47]. Supporting or blending photocatalysts with other materials not only enhances recovery and reduces secondary contamination, but enables recyclability, reusability and increased photocatalyst life span. In addition, hybrid processes involving photocatalysis and membrane technology also enhances the properties of membranes for filtration, adsorption, and other related applications [48]. Trace organic contaminants which include pharmaceuticals and endocrine-disrupting compounds found in raw water, wastewater and sometimes in drinking water can also be effectively treated using photocatalytic membranes [49]. Such contaminants are found in low concentrations of microgram to nanogram per litre. Figure 4 illustrates how a photocatalytic membrane operates during a water treatment process [50].

### 2.1. Photocatalytic NanoMaterials and Electrospun Nanofiber Membranes

**Figure 5:**
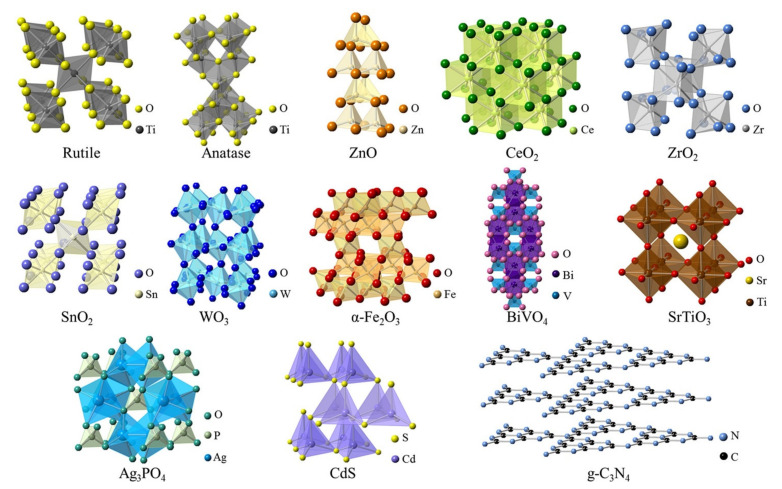
Structures of commonly used photocatalytic materials. Reprinted from [80] with permission from Elsevier.

Electrospun photocatalytic nanofiber materials have excellent properties for various applications because they combine the properties of both the photocatalyst and the nanofibers. Hence, electrospinning of photocatalytic nanomaterials such as ZnO and TiO_2_ with polymers has become popular, either for use as nanocomposites or as photocatalytic nanofibers after removing the polymer by thermal treatment [51,52]. Table 1 shows the properties of various types of photocatalysts that are used for the functionalization or doping of photocatalytic nanofiber membranes. In this review, focus is placed on ZnO and TiO_2_, however other photocatalysts or semiconductors also have intriguing optical properties as shown in Table 1 The corresponding crystal structures of the photocatalysts are shown in Figure 5.

**Table 1:** Crystallographic and electronic properties of common photocatalytic materials.

Photocatalytic Materials	Crystal Structure	*E*_g_ (eV)	λ_e_ (nm)	*E*_CB_ (V vs. NHE)	*E*_VB_ (V vs. NHE)	Ref.
TiO_2_	Tetragonal	3.2	<388	−0.16	+3.04	[53,54,55]
ZnO	Hexagonal	3.4	<388	+0.21	+3.41	[56,57]
CeO_2_	Cubic	3.2	<388	−0.07	+3.13	[58]
ZrO_2_	Monoclinic	5.0	<248	−0.69	+4.31	[59,60]
SnO_2_	Tetragonal	3.5	<354	+0.25	+3.75	[61,62,63]
WO_3_	Monoclinic	2.7	<443	+0.77	+3.47	[64,65]
α-Fe_2_O_3_	Trigonal	2.2	<564	+0.79	+2.99	[66,67]
BiVO_4_	Monoclinic	2.4	<517	+0.49	+2.89	[68,69]
SrTiO_3_	Cubic	3.4	<365	−0.75	+2.65	[70,71,72]
Ag_3_PO_4_	Cubic	2.4	<517	+0.50	+2.90	[73,74]
CdS	Hexagonal	2.4	<517	−0.40	+2.00	[75]
g-C_3_N_4_	2D	2.7	<459	−0.90	+1.80	[76,77,78,79]

**Figure 6:**
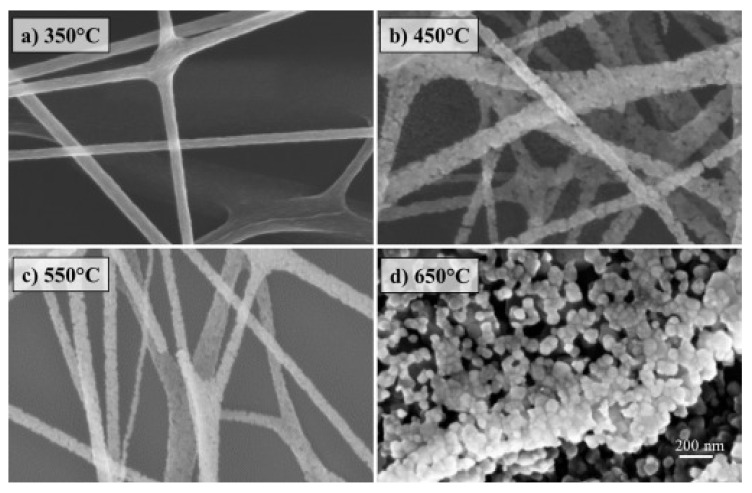
Scanning Electron Microscopy (SEM) images of ZnO nanofibers with diverse morphology produced at different temperatures (**a**) 350 °C, (**b**) 450 °C, (**c**) 550 °C, and (**d**) 650 °C. Reprinted from [83] with permission from Elsevier.

Park et al. and Yang et al. showed that calcination of electrospun ZnO nanofibers can increase the fibers’ diameter and deform their structure [81,82]. Calcination of the ZnO/PVP nanofibers to remove the polymer resulted in an increased diameter of the nanofibers (from 35 to 100 nm) and an increase in surface roughness was also observed [81]. Figure 6 shows an example of electrospun ZnO nanofibers calcined at different temperatures; with increasing temperature, a change in the fiber morphology is perceived. Change is observed from the compact structure and the smooth morphology of the connected nanofibers at 350 °C (shown in Figure 6a), to nanofibers with a rough morphology and nanoparticles beginning to show as they breakaway from each other at 450 and 550 °C (Figure 6b,c), and then to dispersed ZnO nanoparticles at 650 °C (Figure 6d) [83]. This indicates that during the fabrication process, optimization of parameters such as concentrations, temperature, ratios, and viscosity can lead to membranes with the desired physicochemical properties for that specific application.

Electrospinning of TiO_2_ nanofibers for various applications has been well-studied. This includes the work by Chuangchote et al. for the electrospinning of TiO_2_ nanofibers from a solution of titanium (IV) butoxide, poly(vinylpyrrolidone), and acetylacetone. The nanofibers were calcined to remove the solvent and polymer. After calcination at temperatures up to 700 °C, the diameter of the nanofibers was reduced from 409 to 259 nm. Results indicate that the nanofibers contained highly aligned bundled nanofibrils which were beneficial for enhanced crystallinity, large surface area, and higher photocatalytic activity (up to 270 µmol/g of hydrogen evolution using the catalysts calcined at 450 °C) [84]. In contrast to ZnO nanofibers, the calcination of TiO_2_ nanofibers reduced the diameter as a function of temperature because of the removal of the solvent and polymer. Kumar et al. investigated the optical and structural properties of electrospun TiO_2_ nanofibers. They reported that calcined nanofibers appeared to be composed of TiO_2_ grains (~12 nm) packed together to form nanotubes and were responsible for the reduced diameter of up to 60 nm. Optical studies showed a red shift with an increase in the fiber diameter, which is attributed to an increase in the surface stress with a decrease in diameter [85]. 

### 2.2. Coupled and Hybrid Photocatalytic Nanofibers

**Figure 7:**
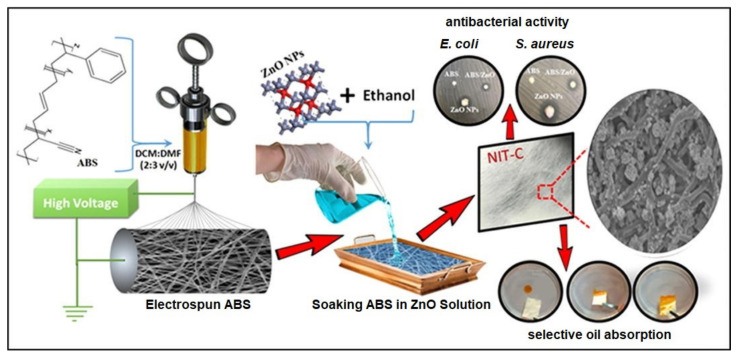
Illustration of the fabrication of ZnO-ABS composite nanofibers membrane with antimicrobial properties tested against *E. coli*, *S. Aureus and selective oil absorption*. Reprinted from [88] with permission from Elsevier.

Coupled photocatalytic nanofibers can also be electrospun to enhance the overall photocatalytic performance. For example, a composite of ZnO-TiO_2_ nanofibers prepared by an electrospinning technique whereby TiO_2_ and ZnO precursors were mixed with PVP polymer solution for electrospinning showed higher photocatalytic degradation of methylene blue compared to TiO_2_ and ZnO alone as reported by Wang et al. [86]. In another study, ZnO-TiO_2_ composite nanofibers prepared by Hwang et al. were used for a bactericidal application. The authors reported excellent antibacterial activity of the electrospun composite nanofibers when tested against Gram-negative *Escherichia coli* and Gram-positive *Staphylococcus aureus.* The tests were carried out under UV and in the absence of light [87]. Manaf et al. conducted a study on the fabrication and performance of poly(acrylonitrile-co-butadiene-co-styrene) [ABS]/ZnO electrospun nanocomposite membranes with oleophilic and antimicrobial properties. The electrospun ABS nanofiber membranes were decorated with floral ZnO nanoparticles using a post-treatment deposition method. The pristine ABS and ZnO-modified nanocomposite membranes showed super oleophilic nature and could selectively separate different oils from the oil–water mixture by a gravity-driven technique with up to 100% separation. The ZnO NPs in the nanofiber could enhance the oil flux and imparted anti-bacterial activity to electrospun ABS membrane against *E. coli* and *S. aureus*. The summary of the fabrication and modification of the ABS nanofiber membranes as well as their antimicrobial activity and water-oil separation are shown on Figure 7 [88].

Hybrid processes based on photocatalysis and membranes have been studied over the past few years to take advantage of the synergy of both processes [89]. Molinari et al. carried out the photocatalytic degradation of azo dyes (Congo red and Patent blue) with a membrane reactor using TiO_2_ as a photocatalyst. The composite membrane was prepared using a conventional phase inversion method. The findings reported after a comparative study between suspended and membrane entrapped TiO_2_ indicated that entrapped TiO_2_ showed satisfactory results. Various parameters such as feed concentration and recirculation rate did not show a major impact on the reaction rates, operating stability, and membrane rejection for both substrate and by-products. The hybrid process showed high photodegradation of Congo red at a high concentration (500 mg/L) under the same conditions as the suspended TiO_2_; this was attributed to the high adsorption induced by the membrane. High permeate flux (30–70 L/m^2^ h) was still maintained upon increasing the concentrations of both dyes up to 500 mg/L; this is attributed to the photodegradation of the dyes on the surface before they foul the membrane [90].

**Figure 8:**
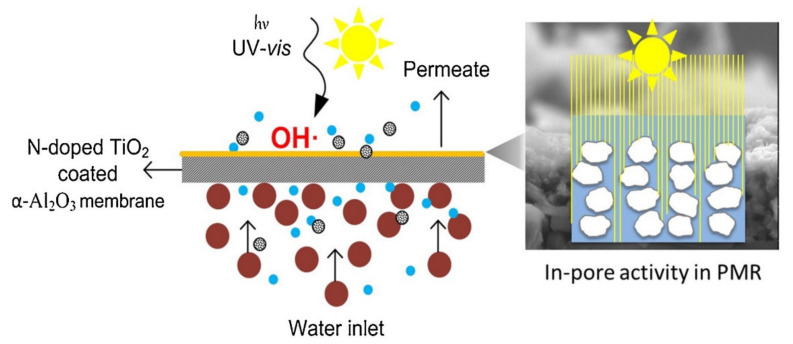
Illustration of simultaneous filtration and photodegradation processes on a photocatalytic-filtration hybrid composite membrane. Reprinted from [92] with permission from Elsevier.

Mendret et al. investigated the influence of pH on the performance of photocatalytic membranes in a dead-end filtration system. Photodegradation and filtration were coupled using TiO_2_/Al_2_O_3_ membranes under UV light in a membrane reactor using Acid Orange as a model pollutant. The nanocomposite membrane was prepared by immobilizing TiO_2_ nanoparticles (mean particles size = 2.5 nm) on alumina membranes using a dip-coating apparatus. Using the TiO_2_/Al_2_O_3_ composite membrane, the flux variation with pH was less pronounced. Due to the TiO_2_ hydrophilic properties, the UV irradiation enabled the stabilisation of the flux. Both pristine and hybrid membranes showed their highest permeate flux at the isoelectric point. Another observation was that the surface of the composite membrane was photoactive, thus allowing membrane fouling mitigation, whereby the efficiency was highly dependent on solution pH, and higher degradation rates were obtained at acidic pH due to the dissociation of Acid Orange and change of TiO_2_ surface properties [91]. Figure 8 illustrates how a photocatalytic nanocomposite membrane simultaneously photodegrades micro-pollutants while filtering. The N-TiO_2_ coated α-Al_2_O_3_ membrane degrades pollutants that are deposited on the surface of the membrane during the filtration process. The N-TiO_2_ layer is the one responsible for the photodegradation process which is activated upon illumination with UV light [92]. 

Fang et al. investigated the effect of photocatalytic oxidation of natural organic matter (NOM) on fouling of low-pressure membranes. In this study, they evaluated the potential of TiO_2_/UV photocatalytic oxidation of natural organic matter to control membrane fouling by coating the membrane with a layer of TiO_2_. The TiO_2_ layer increased the reaction kinetics while a decrease was observed with increasing total organic carbon. At a concentration of 0.5 g/L of TiO_2_, the fouling of both micro- and ultrafiltration membranes was eliminated after 20 min of treatment. Analyses of specific UV absorbance and molecular weight distribution of natural organic matter revealed that the effectiveness in the control of membrane fouling is the result of the changes in molecular characteristics due to the preferential removal and transformation of large, hydrophobic natural organic matter [93].

Table 2 shows polymers from which nanofiber membranes have been prepared, the method of preparation, nanoparticles added and their applications. This information is not limited to the one provided; there are other dozens of peer-reviewed journals, books and reports that used other types of polymers, methods, and nanoparticles. The list (Table 2) shows that a wide range of polymers can be used to fabricate nanofiber membranes whereby PAN [93,94,95,96,97,98,99] and PVP [99,100,101,102] dominate the list. The application of such nanocomposite membranes covers but is not limited to areas such as filtration, photodegradation, and antimicrobial applications [51,103,104,105,106,107,108,109].

In all the studies reviewed in this work, suspended catalysts showed results that were more significant due to the high surface area-to-volume ratio and higher availability of active sites between the catalyst and the pollutants. However, drawbacks such as poor recovery of spent catalysts were observed on suspended catalysts. Overall, the hybrid processes resulted in lower performance, but recovery was increased, and leaching was reduced without compromising the membranes performance. 

## 3. Antimicrobial Membranes

Microorganisms such as bacteria, fungi, archaea, algae, protozoa and viruses [109] form part of the water pollution system and are the cause of various types of waterborne diseases such as polio, malaria, cholera, hepatitis, diarrhea, ascariasis, malnutrition, ringworm and lymphatic filariasis, among others [119,120]. Due to the high levels of water pollution, the high ratio of water demand to water availability, as well as inefficient water treatment facilities, human beings, animals and aquatic biota have to bear the burden of waterborne diseases and infections [121,122,123]. 

**Figure 9:**
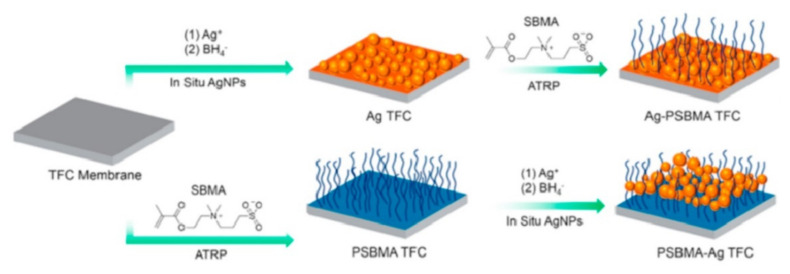
Fabrication process of an antimicrobial TFC membranes used for water treatment. Reprinted from [124] with permission from Elsevier.

Researchers are introducing the use of antimicrobial material in current water treatment methods. Nanocomposites are being produced with the addition of materials that have antimicrobial properties to use in hybrid water treatment processes [124]. Photocatalysis is one of the processes that has been reported to have the ability of “killing” a wide range of bacteria, viruses, algae, endospores, protozoa and fungi, and has also demonstrated the ability to inactivate prions and to destroy microbial toxins [109]. Membrane technology is another process that poses such properties depending on the materials used to fabricate the membrane; however, they are well known to be prone to biofouling. Membrane biofouling is a challenging aspect to control, using chemical, biological, or physical methods due to its compact nature, strong adaptive resistant microbes, and the cost of post-treatment, hence the need for modification [123]. Figure 9 shows two different ways of fabricating a thin film composite membrane with antimicrobial properties that was used for water treatment, as reported by Zhu et al. In the first method, the thin film composite (TFC) membrane is first chemically modified with Ag nanoparticles followed by coating with SBMA. In the second method, the TFC membrane is first co-polymerized with SBMA followed by coating with Ag nanoparticles [124].

**Figure 10:**
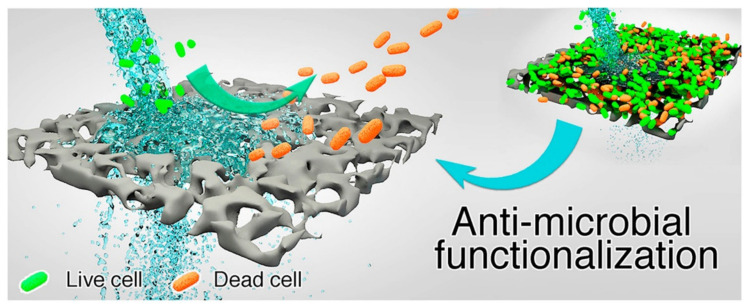
Illustration of antimicrobial activity on a surface of an antimicrobial membrane during water filtration. Reprinted from [124] with permission from Elsevier.

The modification of photocatalytic materials and membrane materials with Ag, Cu or graphene material enhances the antimicrobial properties of the materials which also suggests that such modified materials have self-cleaning/sterilising properties. Figure 10 illustrates how an antimicrobial membrane with cleaning properties operates [124]. This results in improved water quality, enhanced efficiency, and prolonged life span of the membrane by reducing/eliminating fouling [124,125,126]. The properties induced by antimicrobial agents have paved the way for the successful production of antimicrobial photocatalysts, antimicrobial membranes, and antimicrobial-photocatalytic membranes as evidenced by numerous research studies. 

**Figure 11:**
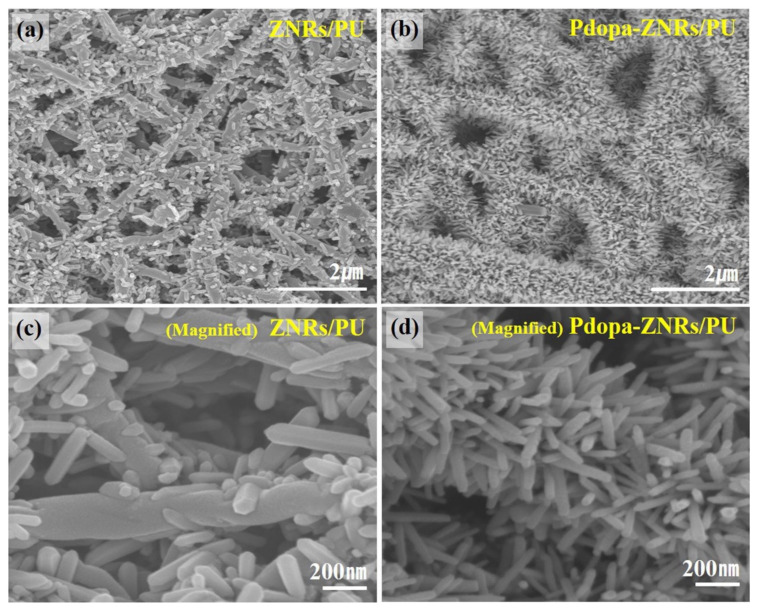
Field emission–scanning electron microscope (FE-SEM) images of (**a**,**c**) ZnO-nanorods/polyurethane, (**b**,**d**) Polydopamine-ZnO-nanorods/polyurethane. Reprinted from [128] with permission from Elsevier.

Zhang et al. fabricated thin-film composite membranes with enhanced antifouling and antimicrobial properties by the incorporation of palygorskite/TiO_2_ hybrid material. Palygorskite and palygorskite/TiO_2_ were embedded on a reverse osmosis polyamide membrane through interfacial polymerisation. The tubular structure of palygorskite played a role in the facilitation of water molecules through the thin-film membrane. The palygorskite-incorporated membranes had a 1.6-fold increase (up to 40 L/m^2^ h) in water flux compared to the bare membranes. The palygorskite/TiO_2_-containing membranes showed a 1.4-fold increase compared to the bare membranes. Antifouling properties were observed with increasing flux against humic acid and bovine serum albumin while antimicrobial properties were also successful against *Escherichia coli* [127]. Hee et al. studied the photocatalytic and antimicrobial activity of ZnO-incorporated electrospun nanofibrous membranes. Polyurethane nanofibers were fabricated by electrospinning followed by coating with polydopamine using the dip-coating method. For the incorporation of ZnO nanoparticles, the polydopamine-coated nanofibers were soaked in a ZnO aqueous solution followed by hydrothermal treatment to grow ZnO nanorods on the surface of the nanofibers. Characterization confirmed that the ZnO nanorods were grown and adhered to the polydopamine-coated polyurethane nanofibers as shown on Figure 11. The resulting material successfully degraded methylene blue within 180 min and showed positive antimicrobial properties against *Escherichia coli* [128]. 

**Figure 12:**
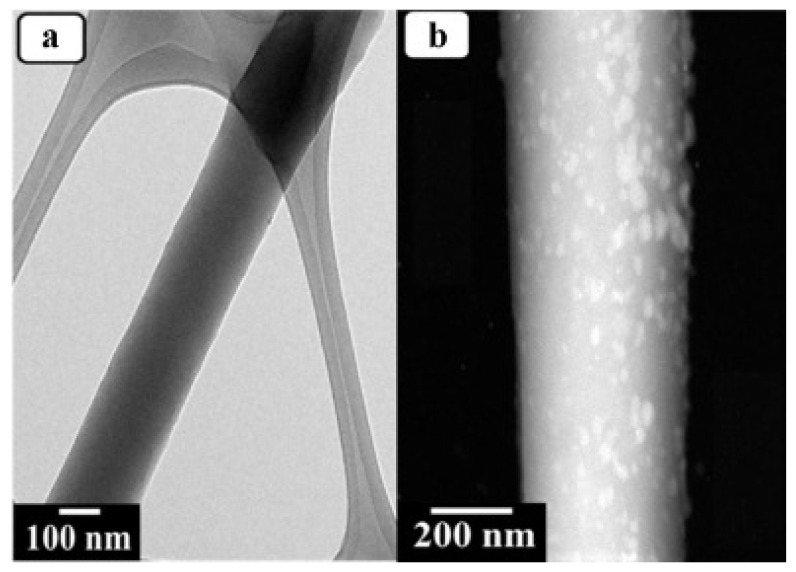
Transmission electron microscopy (TEM) images of (**a**) polyacrylonitrile (PAN) and (**b**) Ag_3_PO_4_/PAN composite membranes used for photodegradation and antimicrobial studies by Panthi et al. Reprinted from [129] with permission from Elsevier.

Panthi et al. produced a photocatalytic and antimicrobial bifunctional composite membrane immobilised with Ag_3_PO_4_ nanoparticles (Figure 12). PAN nanofibers were produced by electrospinning and then modified with amidoxime to use as anchoring sites for Ag^+^ ions; AgNO_3_ was used as the source of Ag. The composite was then reacted with Na_2_HPO_4_ to produce the final bifunctional composite membrane Ag_3_PO_4_/PAN. Antimicrobial activity was confirmed by testing against Gram-negative *Escherichia coli* and Gram-positive *Staphylococcus aureus,* respectively. The composite (150 mg) degraded up to 90% of methylene blue in a solution containing 50 mL of the dye (10 mg/L) within 60 min using a 200 W mercury lamp [129]. Xu et al. fabricated a hybrid antimicrobial nanofiltration membrane. The Ag-Cu_2_O nanowires were prepared by grafting L-dopa on the surface of Cu_2_O nanowires via in situ polymerisation which resulted in a zwitterionic surface suitable for the attachment of Ag^+^ ions. The final Ag-Cu_2_O-PSF composite was fabricated using the in-situ phase inversion method. Antimicrobial studies against *Escherichia coli* and *Staphylococcus aureus* using the composite revealed enhanced antimicrobial activity compared to that of the bare PSF membrane. Bovine serum albumin was used for protein rejection studies whereby the modified membrane rejected up to 94.70% and pure PSF rejected 86.81%. The modified membrane also achieved higher water flux (up to 164.1 L/m^2^·h) compared to the bare membrane (40.4 L/m^2^·h). The modified membranes also demonstrated better flux recovery than the pure PSF membrane [130].

**Figure 13:**
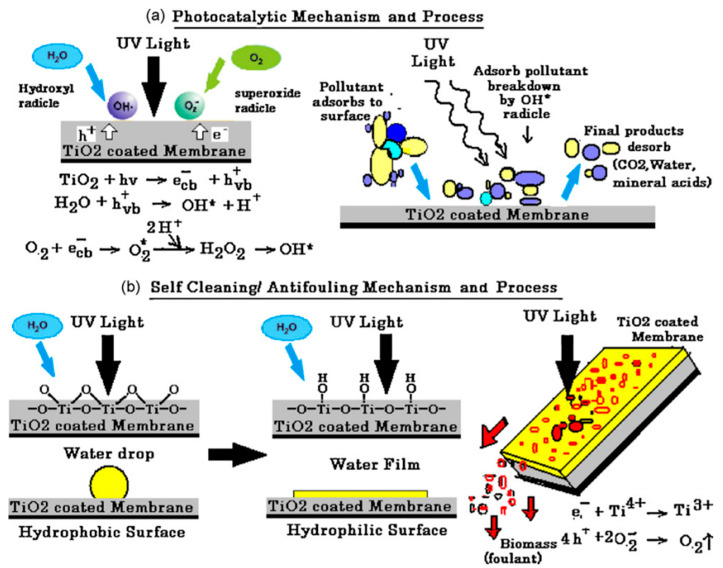
Demonstration of the (**a**) photocatalysis mechanism and process as well as (**b**) Self-cleaning/antifouling mechanism and process of polyvinylidene fluoride (PVDF)/TiO_2_ membrane. Reprinted from [131] with permission from Elsevier.

Damodar et al. studied the self-cleaning, antibacterial, and photocatalytic properties of polyvinylidene fluoride (PVDF) membranes embedded with TiO_2_ (Figure 13). The PVDF/TiO_2_ membranes were prepared via the phase inversion method where the TiO_2_ loading was varied from 0–4%. Generally, PVDF/TiO_2_ membranes showed higher antimicrobial properties compared to the pristine PVDF membrane with 4% TiO_2_ having the highest activity against *Escherichia coli*. Over 95% degradation of Reactive Black 5 was achieved within 60 min using the 2% PVDF/TiO_2_ membrane while the pristine membrane showed no photocatalytic activity. The PVDF/TiO_2_ membranes showed good antifouling and self-cleaning properties under UV irradiation with increased water flux and excellent flux recovery compared to pristine PVDF membranes. The photodegradation and self-cleaning processes on the membrane are as demonstrated on Figure 13 [131]. Jalvo et al. studied the antimicrobial and anti-biofilm efficacy of a TiO_2_ coated glass surface with self-cleaning properties. The material was prepared by coating a glass side with the TiO_2_ suspension. The coated glass slide showed cell reduction viability of over 99% during antimicrobial studies against biofilm-forming bacteria *Staphylococcus aureus* and *Pseudomonas putida.* Self-cleaning properties were tested against the degradation of adsorbed methylene blue. The material achieved 85% degradation confirming that the material has both self-cleaning properties and anti-biofilm efficacy added to their photocatalytic and antimicrobial properties [132]. 

**Figure 14:**
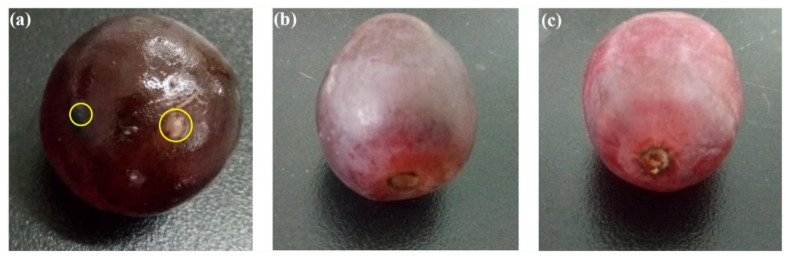
Preservation of red grape packed in different materials at 37 °C for 6 days: (**a**) plastic wrap; (**b**) pure chitosan film; (**c**) chitosan-TiO_2_ film. Reprinted from [133] with permission from Elsevier.

Zhang et al. prepared a chitosan-based antimicrobial film against foodborne pathogens to use in food packaging under visible light. The material was prepared by coating a plastic film-covered glass plate with chitosan-TiO_2_ emulsion crosslinked by epichlorohydrin, followed by drying naturally overnight. The obtained composite film was tested against *Escherichia coli*,* Staphylococcus aureus*,* Candida albicans*,** and *Aspergillus niger*,** and achieved 100% sterilisation within 12 h. Positive results were also obtained in terms of the prevention of microbial growth in packaged red grapes with an extended life span as shown in Figure 14 [133]. Bodaghi et al. studied the photocatalytic antimicrobial effects of TiO_2_ coated packaging film by conducting in vivo and in vitro tests. The TiO_2_-polyethylene film was prepared using the melt blending method whereby modified TiO_2_ powder, polyethylene granules, and glycerol were mixed and blended for an hour. The resulting nanocomposite film was used for all studies. Under in vitro studies, the film reduced the number of surviving cells for *Pseudomonas* spp. and *Rhodotorula mucilaginosa* by 4 and 2 logs compared to 1.35 and 0.64 log reduction of polyethylene, respectively. In vivo studies were conducted on packaged fresh pears under fluorescent light irradiation for 17 days where a significant decrease in mesophilic bacteria and yeast cells was observed for TiO_2_-polyethylene [134].

Antimicrobial activity is one of the most important properties in membrane technology. As indicated, antimicrobial activity prevents microbial membrane fouling which prolongs the lifespan of the membrane [135]. Table 3 shows some of the nanomaterials with antimicrobial activity that can be blended with polymer nanofibers for various applications. Silver nanoparticles are well documented for their excellent antimicrobial activity and it is no surprise that the table shows that pristine and doped-Ag nanoparticles are mostly used for the preparation of antimicrobial nanofiber composite membranes [129,136,137,138,139,140,141,142]. Table 3 further shows that there are other antimicrobial materials such as ZnO, CuO, poly(hexamethylene biguanide) hydrochloride, octadecyldimethyl[3-(trimethoxysilyl)propyl]ammonium chloride, Fe_3_O_4_-COOH, nisin, and metronidazole which can be further explored to reduce the demand of Ag nanoparticles for antimicrobial applications [128,143,144,145,146,147]. The application section of Table 3 indicates that the application of antimicrobial membranes is not limited to water treatment but extends to areas such as cytotoxicity [148,149], drug release [143,146,149], and wound dressing [139,145,148,150,151].

## 4. Other Applications of Hybrid Photocatalytic Membrane Processes

Literature reports show that electrospun nanofiber membranes find application in a wide range of processes within and outside water treatment. It is worth noting that the applicability and efficiency of nanofiber membranes is highly dependent on polymer material intrinsic properties [11]. Therefore, it is imperative to study the properties of the nanofiber membranes prior to application to establish if the membrane is suitable for that specific application. Kaur et al. reviewed the various types of characterization techniques crucial for membranes analysis. These characterization techniques include: atomic force microscopy (surface roughness), scanning electron microscopy (morphology and cross-sectional internal structure), Fourier transform infrared spectroscopy (surface chemistry such as functional groups and bonding nature), tensile test (tensile/mechanical strength and durability), differential scanning calorimetry (thermal properties and rigidity), heat treatment and hot pressing (compactness, integrity and strength), contact angle (hydrophilicity and hydrophobicity) as well as Brunauer, Emmett and Teller (surface area, pore size and pore volume). The review also discusses the liquid intrusion-extrusion techniques for materials to be applied in aqueous solutions [154]. All these techniques form part of the basic analysis of membrane materials and are conventionally used. Other processes whereby photocatalytic and antimicrobial electrospun nanofiber membranes are used include filtration, adsorption and electrocatalysis.

### 4.1. Filtration

Membrane materials are primarily used for filtration processes where the pore size, surface charge, hydrophilicity and shape can be finetuned for suitable filtration processes such as microfiltration, ultrafiltration, nanofiltration, and reverse osmosis. Membrane filtration has gained tremendous attention in recent years as one of the cost-effective and most efficient processes due to stringent guidelines for environmental safety and drinking water quality [155,156]. Even though membrane filtration offers quick and selective filtration for most contaminants, some contaminants such as natural organic matter and pathogens have proven to be challenging to remove via filtration only [157].

**Figure 15:**
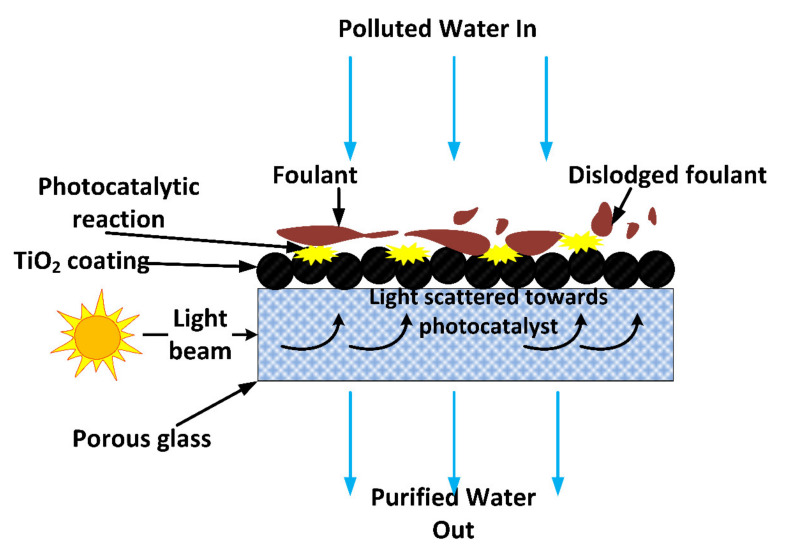
Membrane fouling reduction induced by the addition of TiO_2_ coating through photodegradation. Reprinted from [161].

The properties of membranes can be tuned through the fabrication process, polymer blending, and addition of nanoparticles to overcome such drawbacks. Modification of membranes also plays a significant role in membrane performance and reduced membrane fouling [156] which is a common problem in all membrane processes. Nanoparticles are often incorporated in filtration membrane materials to limit biofouling and prolong the life span. Incorporating nanoparticles in the membrane matrix helps overcome the limitations of separation and leaching of nanoparticles into the aqueous solutions [158,159]. Nanoparticles, especially photocatalytic nanoparticles, have the potential of eliminating the generation of toxic condensates through photodegradation, the generation of toxic condensates being another common problem in membrane filtration [160]. There is ongoing research on hybrid membrane materials which may lead to new and more efficient ways of water treatment which align with drinking water and environmental safety regulations. Figure 15 illustrates the reduction in membrane fouling achieved by coating membranes with photocatalytic materials such as TiO_2_ [161].

Song et al. studied the removal of natural organic matter in aqueous solution using a filtration-photocatalysis integrated method [162]. In this work, PVDF membranes were modified with PEG and TiO_2_ where the PVDF-PEG membranes were compared with PVDF-PEG-TiO_2_ membranes for natural organic matter removal and flux decline. The evaluation of both materials indicated that TiO_2_ modified membranes were more effective in natural organic matter removal and simultaneous reduction of fouling. The membrane also demonstrated good self-cleaning properties under UV IR radiation [163]. Bai et al. fabricated a multifunctional nanocomposite membrane for simultaneous filtration and photodegradation. The nanocomposite consisted of hydrothermally synthesised TiO_2_ nanowires used as a supporting matrix and acid-treated CNT/ZnO nanorods with bridging characteristics. The nanocomposite ultrafiltration membrane integrated both the advantages of photocatalysis and carbon-based nanomaterials with high mechanical strength resulting in efficient production of clean water, high flux, and low fouling potential [108]. Muller at al. reported the preparation and performance assessment of low-pressure affinity membranes for gold nanoparticle filtration based on functionalized electrospun polyacrylates. The study investigated optimized parameters such as concentration, pore size, hydrophilicity, nanofibers size and mechanical strength for optimal pressure drop. The test membranes were prepared by crosslinking copolymers co-polymers methyl methacrylate and 4-methacryloyl-oxy-benzophenone with acrylic acid, n-isopropylacrylamide, 4-vinylpyridine (Pyr), and dimethyldecyl ammoniumethyl methacryl bromide (Nplus) whereby crosslinking was induced by UV irradiation. Their findings showed that the UV-light crosslinking improved mechanical stability and performance properties. The Pyr mechanism was based on physical adsorption while the Nplus mechanism was based on attraction of particles by ionic charge (chemical adsorption). It was further concluded that the pressure drop studies were controlled mostly by the pore size and tensile strength of the nanofiber membranes [164]

**Figure 16:**
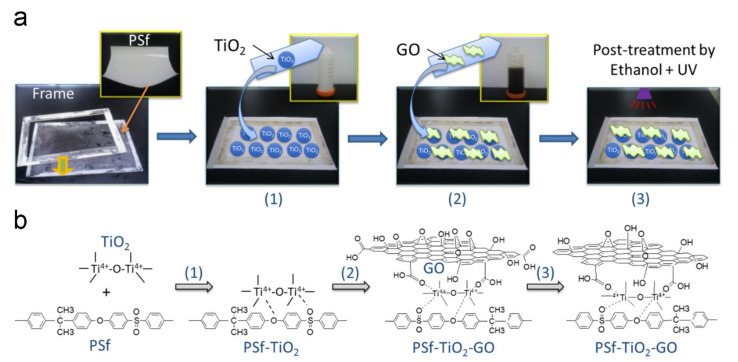
(**a**) Graphical illustration and (**b**) reaction schemes for the surface modification of a polysulfone (PSf) base membrane with TiO_2_–graphene oxide (GO). Reprinted from [165] with permission from Elsevier.

Mendret et al. fabricated a TiO_2_/Al_2_O_3_ hydrophilic composite membrane for separation and photocatalytic degradation of organic pollutants. The 6-layered composite membrane was used in a photocatalytic membrane reactor whereby TiO_2_ coating resulted in enhanced wettability of the Al_2_O_3_ ceramic membrane with high and stable water flux. Flux decline was also reduced during the filtration of Acid Orange 7 [91]. Gao et al. enhanced the photocatalytic performance of a filtration membrane in both UV and visible light irradiation through surface modification using TiO_2_-GO nanocomposites. The surface of a PSf membrane was modified through sequential layer-by-layer deposition of TiO_2_ nanoparticles and GO nanosheets followed by an ethanol/UV post-treatment for the partial reduction of GO as shown on Figure 16. The TiO_2_-GO modified membranes exhibited an increase in photodegradation kinetics of over 80% under UV and four times faster under visible light compared to TiO_2_ and GO modified membranes for the degradation of methylene blue. The membrane flux was also increased while fouling was reduced upon the addition of TiO_2_-GO nanocomposite due to photo-enhanced hydrophilicity and methylene blue degradation [165].

**Figure 17:**
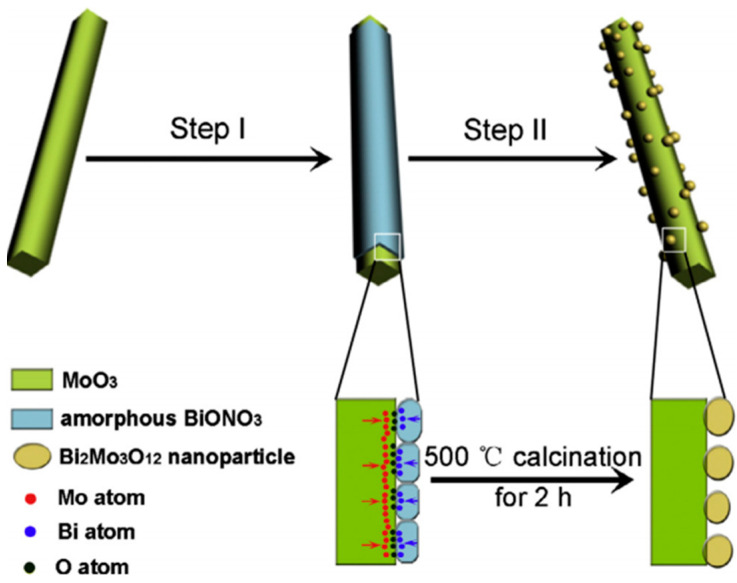
Step-by-step illustration of the fabrication of a Bi_2_Mo_3_O_12_/MoO_3_ nano heterostructure photocatalyst by Liu et al. Reprinted from [166] with permission from Elsevier.

Liu et al. fabricated a MoO_3_ nanowire membrane and a Bi_2_Mo_3_O_12_/MoO_3_ nano-heterostructure photocatalyst for wastewater treatment (Figure 17). Using simulated wastewater, the MoO_3_ nanowire membrane exhibited a high filtration capacity of 1.0 L of methylene blue (50 µM) per gram of MoO_3_ nanoparticles. This membrane can be easily regenerated through 350 °C heat treatment. On the other hand, Bi_2_Mo_3_O_12_/MoO_3_ displayed enhanced photocatalytic degradation of methylene blue under visible light compared to pure Bi_2_Mo_3_O_12_ and MoO_3_ [166]. Molinari et al. conducted a study for the photocatalytic degradation of pharmaceuticals using polycrystalline TiO_2_ and a nanofiltration membrane reactor system. Apart from filtration and photodegradation, adsorption of the substrate onto the catalyst due to the hydrophilic/hydrophobic character of the catalyst was observed. The pure nanofiltration PES membrane achieved a rejection of up to 60% and 30% for furosemide and ranitidine, respectively, in the dark. Degradation using the hybrid membrane reactor indicated that the catalyst was retained by the membrane. Reduced rejection was recorded in the presence of light, oxygen injection, and photocatalyst [167].

Yan Lv et al. fabricated a self-cleaning photocatalytic nanofiltration membrane using a facile biomimetic mineralization process for wastewater treatment. In the fabrication process, a polydopamine/polyethyleneimine layer was fabricated on an ultrafiltration support membrane using a co-deposition method. This was followed by the addition of a photocatalytic layer consisting of β-FeOOH nanorods. The polymeric layer was used as a selective nanofiltration layer and an intermediate layer for anchoring the β-FeOOH nanorods through strong coordination complexes between the catechol groups and Fe^3+^. The photocatalytic membrane exhibited efficient degradation of organic dyes under visible light through the photo-Fenton reaction with the addition of hydrogen peroxide. Under the same conditions, self-cleaning capabilities and effective nanofiltration were observed [168].

The above-mentioned studies and several others demonstrate that hybrid processes between membranes and nanoparticles have tremendous advantages in water treatment such as anti-fouling properties, self-cleaning, selectivity, antimicrobial properties, high flux at low pressures, and offer excellent recovery and reusability of photocatalytic nanomaterials.

### 4.2. Adsorption Technology

Adsorption forms part of the widely used techniques for the treatment of air and water. Adsorbents are fabricated from pristine or modified materials such as polymers, activated carbon, metal organic framework, molecular sieves, zeolites, and other low-cost materials. However, the choice of material depends mostly on the adsorption capacity of the material and the affinity of the material for the target compound [169,170,171]. Adsorption data can be described using either the adsorption diffusion model or the adsorption reaction model. The adsorption diffusion model is based on: (I) external/film diffusion, i.e., diffusion across the liquid media surrounding the adsorbent; (II) internal/intra-particle diffusion, i.e., diffusion in the liquid within the adsorbent’s pores or pore walls; and (III) mass action, i.e., adsorption and desorption between the adsorbate and active sites [172,173].

**Figure 18:**
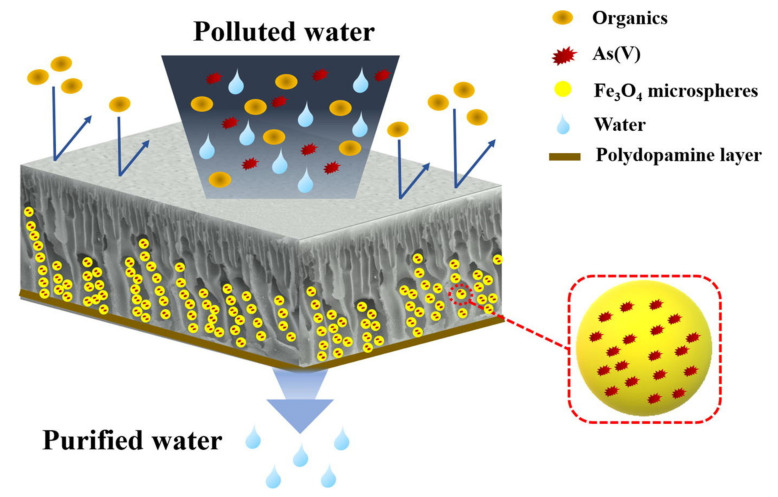
Illustration of adsorption, absorption, and rejection of organic micropollutants and arsenic on the surface and within the membrane pores modified with Fe_3_O_4_ microspheres during a filtration-absorption process. Adapted from [179] with permission from Elsevier.

Adsorption methods are used in water treatment for the removal of phenolic compounds, heavy metals, dye stuff, micropollutants, natural organic matter, microorganisms, and many other water pollutants. These methods are also used for the removal of bad odours and the capture of volatile organic pollutants in wastewater treatment and air purification. Other areas such as medicine, food packaging, upholstery and surface coatings also make use of adsorbents for various reasons [174]. Cross-coupling adsorption materials, addition of nanoparticles and blending with photocatalytic materials can enhance the overall performance of these materials and expand their areas of application [151,175]. Other conditions that can enhance the adsorption capacity of adsorbents include the operating temperature, surface charge of both adsorbent and adsorbate, pH, the concentration of the adsorbent vs. the concentration of the adsorbate, reaction time, and suspended particles [176,177]. All these parameters have to be optimized for every target adsorbate against the adsorbent of choice to establish the efficiency of the adsorbent. Figure 18 illustrates how pollutants are absorbed by a membrane during a filtration process. In this example, arsenic is absorbed within the membrane pores and pore-walls where the Fe_3_O_4_ microspheres responsible for absorption are located. Further, others are adsorbed on the surface of the membrane due to size exclusion, i.e., large molecules remain on the membrane surface (i.e., adsorption and rejection) while smaller molecules go through the pores where they are absorbed. The build-up of larger rejected and adsorbed pollutants on the surface of the membrane results in the formation of a cake which may foul the membrane and decrease its efficiency [178,179]. The build-up of the cake layer and blockage of membranes pores can be reduced through photodegradation and biodegradation by introducing photocatalytic and/or antimicrobial nanoparticles into the membrane matrix. The type of nanoparticle additives will depend on the type of pollutants adsorbed on the membrane surface.

Since some pollutants cannot be efficiently adsorbed due to low affinity to adsorbents, the addition of nanoparticles has been used to enhance the affinity of target materials to adsorbents [180]. Photocatalytic nanomaterials have been blended with adsorbents for either complete degradation of materials with low affinity or partial degradation to by-products that can be easily adsorbed [180,181,182]. Mudhoo et al. reviewed the removal of endosulfan using membrane separation, photocatalytic degradation, bioremediation, and adsorption processes. Endosulfan is a globally used and highly polluting pesticide which cannot be effectively treated using conventional methods. According to Mudhoo et al., successful remediation of endosulfan using any of the above processes has been reviewed and reported with very low efficiency and drawbacks such as: (i) the need for a larger operational area and biomass separation units (bioremediation), (ii) energy cost for large scale photocatalytic degradation (photocatalysis), (iii) production of residual toxic sludge and optimization of operational parameters (adsorption), and (iv) fouling and energy consumption for pressure-driven membranes (membrane technology). However, their findings indicated that hybrid processes have been more efficient compared to single process treatments in terms of cost, simplicity, operability, insensitivity to pollutants and no formation of toxic by-products with to 100% of endosulfan and its derivatives [183]. Zhao et al. conducted a systematic and bibliometric analysis review on the adsorption and photocatalytic nanomaterials for the treatment of emerging contaminants. Their work reviews the nanomaterials used for the adsorption and/or photodegradation of emerging contaminants. Characteristics such as removal capacity, removal mechanism, and influencing factors were discussed. The general conclusion was that as much as the individual processes have their own merits, their hybrid processes demonstrated much better positive performance for the treatment of emerging contaminants. It is indicated that as the adsorbent adsorbs pollutants, it brings them closer (in contact) with the photocatalyst for direct photodegradation; during that process, the photocatalyst cleans up the active sides of the adsorbent to adsorb more pollutants. This simultaneous process together with the free ROS enhances the efficiency of the whole process in terms of cost, time and treatment capacity [184].

**Figure 19:**
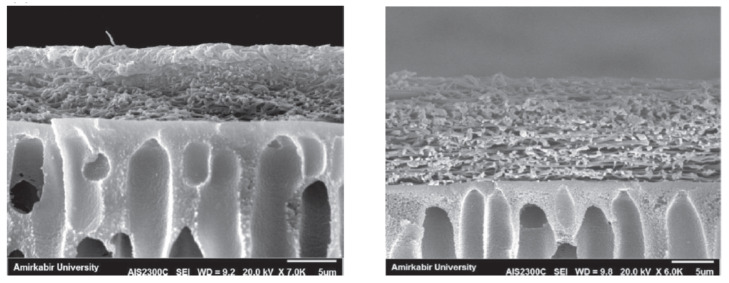
SEM cross-sectional image of chitosan/PVA/PES-a-Fe_3_O_4_ dual layer nanofibrous membranes for adsorption-filtration of Cr(VI) and Pd(II). Reprinted from [181] with permission from Elsevier.

Koushkbaghi et al. reported the fabrication of an adsorptive membrane for the removal of Cr(VI) and Pd(II) ions in aqueous solutions using a dual layer chitosan/PVA/PES nanofibrous membrane filled with animated-Fe_3_O_4_ nanoparticles. Figure 19 shows the SEM cross-section image of the dual layer nanofibrous membrane. The PES support membrane was prepared via a non-solvent induced phase separation method and the PVA/chitosan/a-Fe_3_O_4_ nanofibers were fabricated using electrospinning followed by cross-linking to produce the dual layer nanofibrous membrane. A 2% loading of a-Fe_3_O_4_ onto the dual layer membrane used for both filtration and adsorption yielded the highest recovery of both metal ions with adsorption capacity of 509.7 and 525.8 mg g^−1^ for Cr(VI) and Pd(II), respectively, compared to when the membrane was used for either adsorption or filtration. The purpose of the top nanofiber layer was to evenly disperse the aqueous solution (due to its spongy nature) and slow down the infusion rate through the larger channels of the PES membranes. This step resulted in longer contact time for adsorption to take place while the a-Fe_3_O_4_ increased the affinity of the metals to the membrane [181].

### 4.3. Electrocatalysis

Electrocatalysis is a heterogeneous catalysis process based on electrochemical reactions whereby the reactions occur at the interface of the electrode and electrolyte; the electrode plays both the role of electron acceptor/donor and of the catalyst [185]. The electrocatalytic activity is measured in terms of electrocatalytic kinetics. Electrocatalytic parameters such as overpotential, stability, turnover frequency, Tafel slope, and Faraday efficiency can be used to measure the catalytic activity and efficiency of electrocatalytic reactions [186]. Electrocatalysis plays a critical role in electrochemical reactions such as oxygen reduction reactions, oxygen evolution reactions, hydrogen evolution reactions, emerging CO_2_ reduction reactions, primary alcohol oxidation, liquid fuel conversion devices and other electrochemically related catalytic devices [187,188]. Heterogeneous electrocatalytic reactions require efficient electrocatalysts because they only occur at triple-phase boundary (TPB) regions resulting in sluggish kinetics. A TPB region is a region in which three compounds with different phases interact. In electrocatalysis, a TPB region would be a region where the electrode (in solid form), electrolyte (in liquid form) and dissolved oxygen (in gaseous form) interact [185,187].

**Figure 20:**
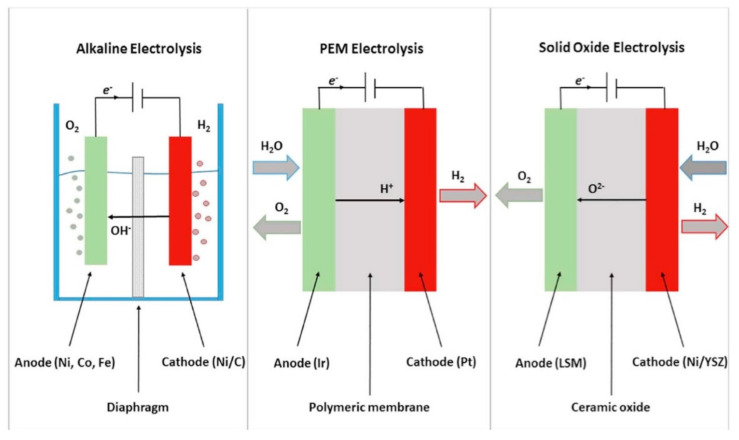
Operating principle of different types of electrochemical electrolysis. Reprinted from [193].

Like most materials in other reaction applications, electrocatalysts are prone to drawbacks such as poor stability, a narrow electrochemical stability window, low specific surface area and poor conductivity, among others [189]. All these drawbacks render the process time consuming, costly and less efficient on its own for large scale applications. As a result, various methods such as doping with nanoporous materials as well as the introduction of pre- and posttreatment methods have been used to enhance the general performance of electrocatalytic processes based on their specific applications [190,191]. Electrocatalysis is one of the most important processes in this era since it finds application in areas such as reduction and oxidation reactions, sensors, electroanalytical chemistry, micro/nanoelectrochemistry, photoelectrochemistry, water and air treatment, electrochemical robotics, medicine, and many others [192]. Figure 20 shows the operating principles of alkaline, polymer-electrolyte membrane (PEM), and solid oxide electrolysis while Table 4 shows the overall reactions at the anode and cathode as depicted on Figure 20.

## 5. Challenges and Future Direction

One of the most significant challenges in water treatment, particularly in the removal of emerging micropollutants, is that conventional methods are not entirely efficient since they were not originally designed for such pollutants and their occurrence at trace levels was not envisaged. In addition, new methods and modification of conventional methods come with drawbacks that make the process costly and time consuming. However, this has often been overcome by means of coupled processes that aim at eliminating drawbacks associated with each process and enhance the overall efficiency of the new process as demonstrated earlier in this review.

The excellent performance properties of nanoparticle-infused nanofiber membranes in various processes for water treatment should be used as a motivation to explore and expand the use of these nanocomposite materials within and outside the water treatment arena. Therefore, the use of these specialized nanocomposites should not be limited to water application. Fields such as drug delivery, wound healing, air purification, surface coating, food packaging, clothing, energy, automotive and personal protective clothing, among others, offer a great platform for application of such nanocomposite materials in a quest to diversify their use. Therefore, the use of compatible materials and processes that complement each other when fabricating a coupled nanofiber composite for a specific application should be considered.

## 6. Conclusions and Perspectives

This review discussed extensive studies conducted in the field of nanoparticle-infused nanofiber membranes with particular emphasis on their applications in photocatalysis, antimicrobial fields, and water remediation. Tremendous progress that has been attained in these fields including fabrication, properties and performance, has been thoroughly discussed. Studies conducted in the field under review have demonstrated that enhanced efficiency has been achieved for the removal of micropollutants in water and in other related applications. Supporting nanoparticles onto nanofibers, polymeric membrane matrices, electrodes, ceramics, and many others has been shown to reduce leaching and the loss of photocatalytic activity during application in aqueous solutions. This also enhances the recyclability and reusability of the photocatalysts.

Due to their tremendous performance, photocatalytic and antimicrobial materials have been coupled with other processes such as membrane filtration, adsorption and electrochemical processes for air and water treatment applications. Considering the excellent work that has been demonstrated on various types of photocatalytic nanomaterials to produce nanofiber composite membranes thus far, further improvements should be explored for enhanced performance, stability, and reusability. Scaling up and commercialization of these excellent nano-infused nanofiber materials for practical applications can be explored once a thorough assessment has been made on the fate, toxicity, and cost of these nanofiber composite membranes.

## Figures and Tables

**Table 2:** Photocatalytic nanofiber membranes, their preparation methods, and applications.

Polymer/Membrane	Additive	Method	Application(s)	Photocatalytic Efficiency (%)	Ref.
Polyvinylpyrrolidone	Ag/TiO_2_	Co/Blend-Electrospinning	Filtration Photodegradation (Methylene blue) Antimicrobial (*E. coli*)	80	[100]
Polyvinylpyrrolidone	TiO_2_	Electrospinning	Photodegradation (Rhodamine b)	72	[101]
Polyvinylpyrrolidone	TiO_2_/C	Electrospinning	Capture and photocatalytic conversion of particulate matter	99.92	[102]
Polyacrylonitrile	ZnO/TiO_2_	Electrospinning	Photodegradation (Malachite green)	99	[39]
Polyacrylonitrile	biogenetic silica	Electrospinning	Photodegradation (malachite green)	100	[94]
Polyacrylonitrile	SiO_2_-TiO_2_-NH_2_	Electrospinning	Photodegradation (Malachite green and Acid red 27)	100	[95]
Polyacrylonitrile	Ag/AgCl	Electrospinning	Filtration Photodegradation (Methyl orange)	85	[96]
Polyacrylonitrile	ZnO/Ag	Electrospinning—reflux	UV-shielding Photocatalysis (Methylene blue) Antimicrobial (*S*. *aureus*)	99	[97]
Polyacrylonitrile	TiO_2_/MOF/CNT	Electrospinning—self assembly	Photodegradation (Hydrogen sulphide)	93.5	[98]
Polyacrylonitrile	α-Fe_2_O_3_/rGO	Hydrothermal vacuum filtration	Photodegradation (Methylene blue)	98.5	[99]
Polyaniline	TiO_2_/SiO_2_	Electrospinning	Photodegradation (Methyl orange)	87	[51]
Free standing	CNTs/TiO_2_	Chemical vapor deposition	Photodegradation (Methylene blue)	Not specified	[103]
Free standing	CNT/ZnO/TiO_2_	Hydrothermal	Filtration Photodegradation (Acid Orange 7) Adsorption (Acid Orange 7)	100	[108]
Free standing	Zr-TiO_2_	Electrospinning	Photodegradation (Methylene blue)	95.4	[110]
Nylon	ZnO	Electrospinning—atomic layer deposition	Photodegradation (Rhodamine b)	99	[104]
Chitosan	Algae-TiO_2_/Ag	Electrospinning	Photocatalytic reduction of Cr(VI)	91	[105]
Graphene oxide	ZnO	Vacuum filtration	Filtration Anti-biofouling via photodegradation (Powder milk and direct red 16 dye)	90.5	[107]
Polyether sulfone	ZnO/MWCNTs	Non-solvent induced phase inversion	Filtration Antimicrobial (*E. coli*) Photodegradation rejection (Rhodamine b)	99.6	[106]
Polyimide	ZnO	Electrospinning	Photodegradation (Methylene blue)	98	[111]
Cellulose acetate/polyurethane	ZnO	Solution dispersion blending	Photodegradation (Reactive Red 11 and Reactive Orange 84)	100 95	[112]
Cellulose	TiO_2_-coreshell	Vacuum filtration	Photodegradation (methyl orange)	100	[113]
Polyurethane	Ag-TiO_2_	Electrospinning	Photodegradation (Dairy effluents)	95	[114]
PAN-Alumina hollow fiber	GCN	Electrospinning	Oilfield produced water treatment.	99	[115]
Nylon-6	TiO_2_	Electrospraying and electrospinning	Photodegradation (Methylene blue) Toxicity control of chlorophenols	100	[116]
Polyvinylidene fluoride	Sm-ZnO	Electrospinning	Photodegradation (Reactive golden yellow and Rhodamine b)	100	[117]
Polyvinylidene fluoride—Polyacrylonitrile	TiO_2_	Electrospinning	Photodegradation (Rhodamine b) Oil-water separation	97 99	[118]

**Table 3:** Antimicrobial nanofiber membranes and their various applications.

Polymer	Antimicrobial Agent	Method	Application	Antimicrobial Activity	Ref.
Polyurethane	Polydopamine-ZnO	Electrospinning	Antimicrobial (*E. coli)* Photodegradation (Methylene blue)	Active	[128]
Polyacrylonitrile	Ag_3_PO_4_	Electrospinning	Antimicrobial (*E. coli* and *S. aureus)* Photodegradation (Methylene blue)	Active	[129]
Polyacrylonitrile	Ag nanoparticles	Electrospinning	Antimicrobial (*E. coli* and *S. aureus)*	Active	[136]
Polyacrylonitrile	Ag nanoparticles	Electrospinning	Antimicrobial (*E. coli* and *S. aureus*)	Active	[137]
Polyacrylonitrile	Ag nanoparticles	Electrospinning	Antimicrobial (*E. coli* and *S. aureus*) Forward osmosis	Active	[138]
Chitosan	Ag nanoparticles	Centrifugal spinning	Antimicrobial (*S. aureus)* Wound healing	Active	[139]
Polysulfone	CNT/Ag	Radical solution polymerization and wet-phase inversion	Antimicrobial (*E. coli* and *B. subtilis*)	Active	[140]
3D woven fabric filters	Ag nanoparticles	Electrospinning	Antimicrobial (*S. aureus)* Water treatment	Active	[141]
Polyvinyl alcohol	Polyimide-Ag	Electrospinning	Antimicrobial (*E. coli* and *S. aureus*) Oily wastewater treatment	Active	[142]
Polyacrylonitrile	CuO	Electrospinning	Antimicrobial (*E. coli* and *B. subtilis)* for breath masks Drug release	Active	[143]
Chitosan/poly(ethylene oxide)	Poly(hexamethylene biguanide) hydrochloride	Electrospinning	Antimicrobial (*E. coli* and *S. aureus*)	Active	[144]
Poly(ε-caprolactone) and gelatine	Octadecyldimethyl[3 -(trimethoxysilyl)propyl]ammonium chloride	Electrospinning	Antimicrobial *(S. aureus* and *P. aeruginosa)* Wound dressing	Active	[145]
Polylactic acid	Fe_3_O_4_-COOH	Electrospinning	Antimicrobial (*E. coli* and *S. aureus)* Drug delivery	Active	[146]
Triaxial	Nisin	Electrospinning	Antimicrobial *(S. aureus)*	Active	[147]
Cellulose acetate/polyester urethane	Polyhexamethylene biguanide	Electrospinning	Antimicrobial (*E. coli*) Cytotoxicity Wound healing	Active	[148]
Polycaprolactone/gelatine	Metronidazole	Electrospinning	Antimicrobial (*F. nucleatum*) Cytotoxicity (L929 Cells) Drug delivery	Active	[149]
Silk fibroin	Peptide motif	Electrospinning	Antimicrobial *(S. aureus*, *E. coli*, *S. epidermidis* and *P. aeruginosa)* Wound dressing	Active	[150]
Polycaprolactone	2-(Methacryloyloxy) ethyl trimethylammonium/polycaprolactone	Cross-linking polymerization and electrospinning	Antimicrobial (*E. coli* and *S. aureus*) Wound dressing	Active	[151]
Nylon 6	*N*-Halamine	Electrospinning	Antimicrobial (*E. coli* and *S. aureus)*	Active	[152]
Polycaprolactone	Peptide dissolved micro needles	Coaxial electrospinning and electrospray deposition	Antimicrobial (*S. aureus*, *K. pneumoniae*, *A. baumannii*, and *P. aeruginosa*) Chronic wound dressing	Active	[153]

**Table 4:** Overall reactions at the anode and cathode with different electrolytic reactions [193].

Electrolysis	Overall Reaction at Anode	Overall Reaction at Cathode
Alkaline	4OH^−^ → 2H_2_O + 4e^−^ + O_2_	4H_2_O + 4e^−^ → 4OH^−^ + 2H_2_
Polymer-electrolyte membrane	2H_2_O → 4H^+^ +4e^−^ + O_2_	4H^+^ + 4e^−^ → 2H_2_
Solid oxide	O^2^ → ½ O_2_ + 2e^−^	H_2_O + 2e^−^ → H_2_ + O^2−^

## Data Availability

Not Applicable.
